# Soluble immune-checkpoint factors: a potential immunotherapy biomarker

**DOI:** 10.1172/JCI179352

**Published:** 2024-04-01

**Authors:** Aaron C. Tan, Sarah L. Cook, Mustafa Khasraw

**Affiliations:** 1 Division of Medical Oncology, National Cancer Centre Singapore, Singapore.; 2 Duke-NUS Medical School, National University of Singapore, Singapore.; 3 The Duke Cancer Institute, Duke University Medical Center, Durham, North Carolina, USA.

## Abstract

There is unmet need for additional biomarkers to better select patients with non–small cell lung cancer (NSCLC) that are likely to benefit from immunotherapy in order to improve patient outcomes, reduce patient toxicity, and relieve the growing burden of healthcare costs. In this issue of the *JCI*, Hayashi and colleagues evaluated soluble forms of the immune checkpoint molecules PD-L1, PD-1, and CTLA-4 in the plasma of patients with advanced NSCLC who had been treated with anti-PD-1/L1 therapy. The findings suggest that these soluble immune-checkpoint factors may provide a complementary biomarker to PD-L1 IHC, although application into the clinic may not be straightforward.

## Abstract 2

There is unmet need for additional biomarkers to better select patients with non-small cell lung cancer (NSCLC) that are likely to benefit from immunotherapy in order to improve patient&rsquo;s outcomes, reduce patient toxicity, and relieve the growing burden of healthcare costs. In this issue of the <em>JCI</em>, Hayashi and colleagues evaluated soluble forms of the immune checkpoint molecules PD-L1, PD-1, and CTLA-4 in the plasma of patients with advanced NSCLC who had been treated with anti-PD-1/L1 therapy. The findings suggest that these soluble immune-checkpoint factors may provide a complementary biomarker to PD-L1 IHC, although application into the clinic may not be straightforward.

## Current status for ICI biomarkers in NSCLC

In nononcogene-addicted metastatic non–small cell lung cancer (NSCLC), immune checkpoint inhibitor (ICI) therapy is now standard of care in the first-line setting (1). Treatment is commonly stratified by PD-L1 IHC testing, to select between anti-PD-1/L1 monotherapy, combination with chemotherapy, or anti-CTLA-4 therapy. However, PD-L1 IHC is an imperfect biomarker, with negative PD-L1 not precluding a response to ICI therapy and notable rates of nonresponse despite high PD-L1 levels (2). Further, appropriate implementation and interpretation can be challenging, with multiple antibody clones and platforms or assays available and tumor heterogeneity presenting prominent areas of concern (3). Consequently, there is unmet need for additional biomarkers to better select patients likely to benefit from ICI therapy, and therefore to improve patient’s outcomes, reduce patient toxicity, and relieve the growing burden of healthcare costs.

## Soluble forms of immune checkpoint molecules

In this issue of the *JCI*, Hayashi and colleagues (4) evaluated soluble forms of the immune checkpoint molecules PD-L1, PD-1, and CTLA-4 in the plasma of patients with advanced NSCLC who had been treated with anti-PD-1/L1 ICI therapy. Using prospective (discovery) and retrospective (validation) cohorts of patients treated with anti-PD-1/L1 therapy, they found that high concentrations of these soluble factors may be associated with hyper or terminal exhaustion of antitumor immunity. The ability of the soluble factors to stratify tumors was improved when combined with tissue that labeled positive for PD-L1 with IHC, especially that with a tumor proportional score (TPS) for PD-L1 expression of 50% or more. Therefore, these factors could potentially function as a complementary biomarker and identify patients unlikely to respond.

Despite these encouraging findings, there are several concerns with the robustness of the study results. There were substantial differences between cohorts, with the validation cohort being retrospective, having a higher number of smokers, lower rates of *EGFR* mutations, and a proportion with unknown tissue PD-L1 expression status. There is increasing evidence for the use of targeted therapies against a range of oncogenic drivers, even more so in patients of Asian descent (5). There is an unclear role for immunotherapy in many of these oncogene-driven subsets, casting doubt on the current study’s patient population. The overall numbers of patients were also small, and for important subgroups, such as the presence of 2 favorable factors with tumor PD-L1 labeling 50% or more in the validation cohort, the patient number was as low as 3. Crucially, this study was also limited to patients treated with anti-PD-1/L1 monotherapy, and the studied cohorts may not represent current standards of care. Typically, anti-PD-1/L1 monotherapy may be given to treatment-naive patients with a PD-L1 TPS score of 50% or greater (or less commonly equal to or greater than 1%–49%); however, treatment naive patients were only studied in a small proportion of patients belonging to the validation cohort. Patients treated with combined anti-PD-1/L1 therapy with chemotherapy were also not evaluated in this study, and extrapolation of findings to this group of patients is difficult.

There is, however, emerging data to suggest that soluble immune checkpoint molecules, particularly soluble PD-L1, may be a prognostic biomarker for immunotherapy as evidenced by a recent metaanalysis (6). The precise biological mechanism for this possibility remains to be elucidated. Observations in the Hayashi et al. study that soluble PD-1 may be derived from peripheral exhausted CD8^+^ T cells positive for PD-1 provides some useful insights into the potential correlation with terminal exhaustion (4). The exact source(s) of these soluble immune checkpoint molecules however, is also incompletely understood (7). Consequently, deeper immunoprofiling and orthogonal validation are needed to confirm these hypotheses and gain a better understanding of their role in predicting response to ICI therapy.

## Numerous efforts to develop biomarkers with limited success

Despite the need for improved biomarkers over PD-L1 IHC, the development of additional and alternative biomarkers has been fraught with barriers. Tumor mutational burden (TMB) in either tissue or blood for example, has been shown to enrich for patients likely to respond (8). However, a lack of assay standardization and overall limited evidence to adopt this complex biomarker have restricted its application into the clinic for NSCLC (9). Other prominent examples include RNA expression profiles such as the T-cell inflamed GEP score (10, 11). Various other assessments of neoantigens, genetic and epigenetic signatures, immune microenvironment by IHC or transcriptomics, and the microbiome, are all in various stages of development with varying data (12). Fundamentally however, these biomarkers have limited predictive power for patient response to ICI therapy and are therefore inadequate for patient stratification in real-world clinical practice (13, 14). Consequently, whether soluble immune-checkpoint factors offer improvement over many of these other biomarkers remains questionable.

Additional considerations for emerging biomarkers include logistic and technical limitations. Technical validation would need to be performed across different patient populations. The costs of assay, appropriate turnaround time, adequate clinician interpretation, and understanding of results, will all impact the ability to implement any novel assay into routine clinical practice. A plasma biomarker, using liquid biopsy, does have advantages such as being a noninvasive tool with greater patient acceptance and easier sample processing (15). Finally, regulatory considerations pose an important hurdle to overcome, and large prospective trials incorporating a candidate biomarker would need to be conducted. The widespread applicability of the chemiluminescent magnetic technology (HISCL system) used in Hayashi et al. (4) may be a concern.

## Future directions for research into ICI therapy biomarkers

**Figure 1:**
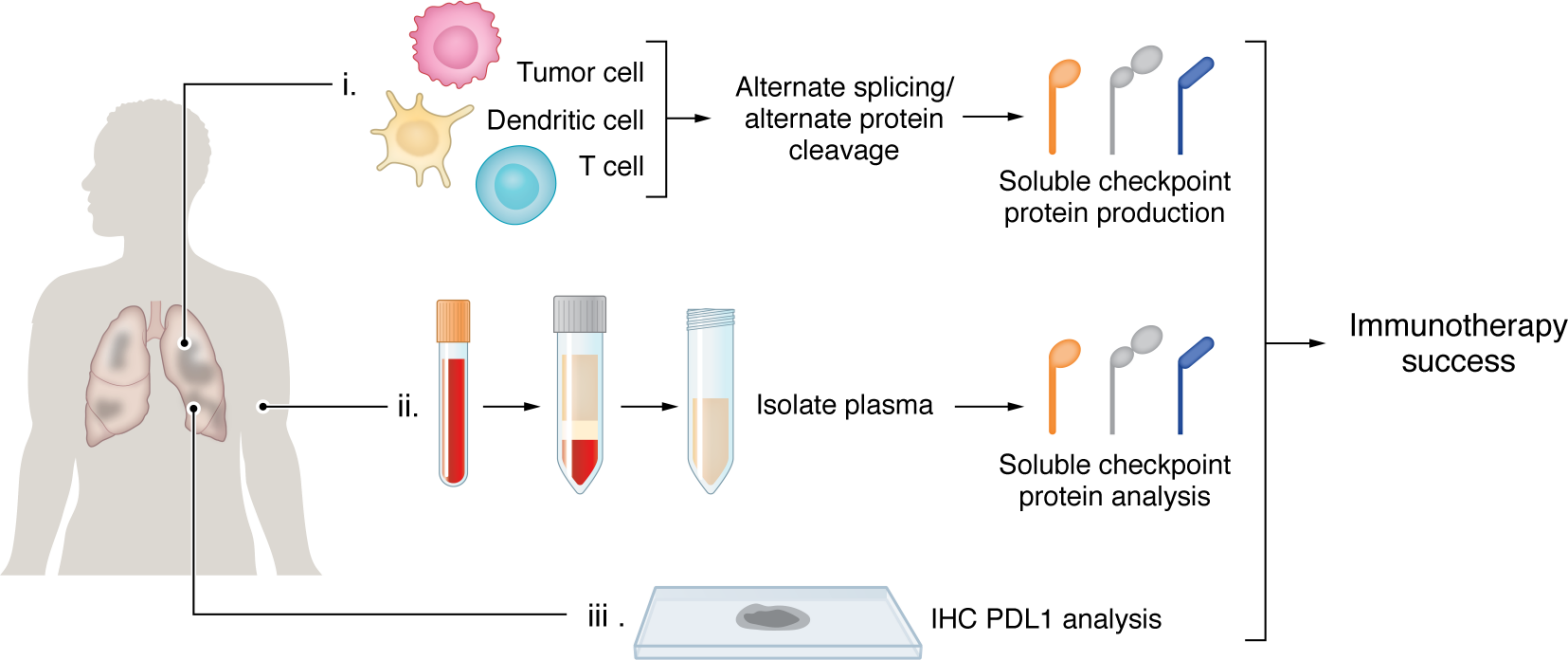
Tissue analysis to determine the success of immunotherapeutic intervention may require multiple analyses. Determining whether immunotherapeutic intervention administered to NSCLC patients will be successful may require multiple analyses on different tissue specimens. (i) Tumor samples may be tested via microfluidic assays that detect splicing variants of immune checkpoint proteins produced by tumor cells and immune cells such as dendritic or T cells (7). (ii) Soluble checkpoint proteins can be detected in the plasma of patients (4). (iii) Tumor specimens may be assessed histologically for immune checkpoint proteins.

Despite the difficulties in developing an effective biomarker, there remains promise that technologies such as liquid biopsies, spatial transcriptomics and single-cell -omics may enhance our ability to translate our biologic understanding of NSCLC into practical clinical implementation (Figure 1). In addition, data driven approaches, with integration of broad -omic profiling and artificial intelligence (AI), may allow for multivariate approaches. As Hayashi, Chamoto and colleagues (4) suggest, soluble immune-checkpoint factors may be more complementary rather than a replacement for PD-L1 IHC. However, the integration of additional layers of insight into antitumor immunity are likely needed before any such assay is ready to enter the clinic. The complexity of the interaction between the immune system and tumor cells suggests improving predictive power may require global assessment of multiple biomarkers (12). As emerging immunotherapies enter the clinic, biomarkers may also need to account for varied drug mechanisms of action and combination therapies, illustrating the complexities and difficulties in biomarker development.

## Version 1 04/01/2024

Electronic publication
